# Biosynthesis of Novel Tellurium Nanorods by *Gayadomonas* sp. TNPM15 Isolated from Mangrove Sediments and Assessment of Their Impact on Spore Germination and Ultrastructure of Phytopathogenic Fungi

**DOI:** 10.3390/microorganisms11030558

**Published:** 2023-02-22

**Authors:** Mohamed N. Abd El-Ghany, Salwa A. Hamdi, Shereen M. Korany, Reham M. Elbaz, Mohamed G. Farahat

**Affiliations:** 1 Botany and Microbiology Department, Faculty of Science, Cairo University, Giza 12613, Egypt; 2 Zoology Department, Faculty of Science, Cairo University, Giza 12613, Egypt; 3 Department of Biology, College of Science, Princess Nourah bint Abdulrahman University, P.O. Box 84428, Riyadh 11671, Saudi Arabia; 4 Botany and Microbiology Department, Faculty of Science, Helwan University, Cairo 11795, Egypt; 5 Department of Biology, College of Science, University of Bisha, P.O. Box 551, Bisha 61922, Saudi Arabia; 6 Biotechnology Department, Faculty of Nanotechnology for Postgraduate Studies, Sheikh Zayed Branch Campus, Cairo University, Sheikh Zayed City 12588, Egypt

**Keywords:** tellurium nanoparticles, green synthesis, biogenic nanoparticles, phytopathogenic fungi, antifungal activity

## Abstract

The biosynthesis of nanoparticles using green technology is emerging as a cost-efficient, eco-friendly and risk-free strategy in nanotechnology. Recently, tellurium nanoparticles (TeNPs) have attracted growing attention due to their unique properties in biomedicine, electronics, and other industrial applications. The current investigation addresses the green synthesis of TeNPs using a newly isolated mangrove-associated bacterium, *Gayadomonas* sp. TNPM15, and their impact on the phytopathogenic fungi *Fusarium oxysporum* and *Alternaria alternata*. The biogenic TeNPs were characterized using transmission electron microscopy (TEM), scanning electron microscopy (SEM), energy-dispersive X-ray spectroscopy (EDX), X-ray diffraction (XRD), Raman spectroscopy and Fourier transform infrared (FTIR). The results of TEM revealed the intracellular biosynthesis of rod-shaped nanostructures with a diameter range from 15 to 23 nm and different lengths reaching up to 243 nm. Furthermore, the successful formation of tellurium nanorods was verified by SEM-EDX, and the XRD pattern revealed their crystallinity. In addition, the FTIR spectrum provided evidence for the presence of proteinaceous capping agents. The bioinspired TeNPs exhibited obvious inhibitory effect on the spores of both investigated phytopathogens accomplished with prominent ultrastructure alternations, as evidenced by TEM observations. The biogenic TeNPs impeded spore germination of *F. oxysporum* and *A. alternata* completely at 48.1 and 27.6 µg/mL, respectively. Furthermore, an increase in DNA and protein leakage was observed upon exposure of fungal spores to the biogenic TeNPs, indicating the disruption of membrane permeability and integrity. Besides their potent influence on fungal spores, the biogenic TeNPs demonstrated remarkable inhibitory effects on the production of various plant cell wall-degrading enzymes. Moreover, the cytotoxicity investigations revealed the biocompatibility of the as-prepared biogenic TeNPs and their low toxicity against the human skin fibroblast (HSF) cell line. The biogenic TeNPs showed no significant cytotoxic effect towards HSF cells at concentrations up to 80 μg/mL, with a half-maximal inhibitory concentration (IC_50_) value of 125 μg/mL. The present work spotlights the antifungal potential of the biogenic TeNPs produced by marine bacterium against phytopathogenic fungi as a promising candidate to combat fungal infections.

## 1. Introduction

The antimicrobial activity of various nanomaterials has gained increasing interest owing to their diverse medical, agricultural and environmental applications. Undoubtedly, nanoparticles have extraordinary advantages compared to bulk materials due to their larger surface-to-volume ratios and higher spatial confinement [1]. In this trend, extensive attempts have been employed to fabricate metal, metal oxide and metalloid nanoparticles as alternative antimicrobial agents, such as silver, gold, copper, zinc, selenium, and tellurium nanoparticles [2,3,4,5,6,7,8,9,10,11]. Recently, tellurium nanoparticles (TeNPs) have drawn significant attention due to their antibacterial and antifungal activity, besides biocompatibility [12]. Tellurium is a toxic rare element metalloid that belongs to chalcogens, with intermediate properties between metals and non-metals [13]. In a recent approach, the employment of the high efficiency of some tellurite-resistant microorganisms to biomineralize the metalloid oxyanions to less toxic elemental TeNPs resulted in a reduction in tellurium toxicity and increased its biocompatibility [14].

Green nanotechnology employs natural biological resources such as bacteria, fungi, algae and plants to achieve an eco-friendly cost-effective fabrication of nanoparticles [15,16,17]. The ability of these organisms to act as nanofactories for the biosynthesis nanoparticles makes them an excellent green alternative to chemical synthesis, avoiding the use of harsh and toxic chemicals. In this regard, many TeNPs-producing microorganisms have been isolated from diverse environments and have been reported to synthesize various shapes of TeNPs, such as a pillar, needle, spherical, cube and rod-shaped nanoparticles [18,19,20,21]. It has been reported that tellurite-biomineralizing bacteria produce black precipitates within and/or outside the cells as a common feature for intracellular and extracellular production of TeNPs [22,23,24]. In extracellular biosynthesis, the added tellurite salts are reduced into TeNPs in the culture broth, whereas, in the intracellular process, the biosynthesis of TeNPs occurs inside the cells, followed by cell lysis to be recovered and purified [25,26,27,28]. It has been believed that mangrove sediments contain numerous microbial groups that play vital roles and environmental processes in the mangrove ecosystem. Indeed, mangrove sediments-derived microbes have been supposed as a rich reservoir of natural product diversity and contribute in biomineralization and biotransformation of minerals [29,30,31,32,33,34,35,36]. Though tellurite-reducing bacteria were isolated from various terrestrial and marine habitats [37,38,39,40,41], their isolation from mangrove ecosystems is relatively unexplored.

Today, extensive losses of crops at the global level are caused by fungal infections, especially with the continuous emergence of fungicide resistance. It has been thought that phytopathogenic fungi are responsible for losing up to 20% of the global crop yields yearly, and about 10% of crops are destroyed due to post-harvest fungal infections [42]. The phytopathogen *Fusarium oxysporum* is a ubiquitous soilborne fungal pathogen that ranks 5th among the top 10 fungal plant pathogens, and was reported to cause devastating vascular wilt in more than 100 plant species [43]. Besides being a phytopathogen, *F. oxysporum* has also emerged as a human pathogen [44,45,46]. Additionally, *Alternaria alternata* is an important fungal pathogen that has a wide host range, causing considerable losses in the yield of many crops [47,48,49,50]. Like *F. oxysporum*, the fungal pathogen *A. alternata* has been documented as a causal agent of human infections [51,52,53]. The antimicrobial activity of TeNPs against human bacterial pathogens is well-documented. However, little data unveiling the antifungal activity of TeNPs against phytopathogens is available. In a recent study, citric juice-mediated TeNPs exhibited antibacterial effects against multidrug-resistant *Escherichia coli* and methicillin-resistant *Staphylococcus aureus* [54]. TeNP-induced antibacterial activity has been attributed to superoxide-mediated oxidative stress, leading to cytoplasmic thiol oxidation and inactivation of the iron–sulfur center-containing enzymes as well as the peroxidation of the membrane lipids, causing cell death [55,56]. In addition, biogenic TeNPs produced by *Ochrobactrum* sp. MPV1 exerted antibacterial and biofilm eradication activity against *Escherichia coli* JM109, *Pseudomonas aeruginosa* PAO1, and *Staphylococcus aureus* ATCC 25923 that was associated with the production of reactive oxygen species [57]. On the other hand, a limited number of published articles demonstrate the antifungal activity of TeNPs against yeasts and filamentous fungi. The antifungal activity of biogenic TeNPs against *Candida albicans* ATCC14053 is assumed to be due to the inhibition of squalene monooxygenase, which is a rate step-limiting enzyme in the ergosterol and cholesterol biosynthesis pathway involved in the synthesis of ergosterol, cholesterol, and phytosterols [58]. Herein, we report for the first time on the facile biosynthesis and characterization of TeNPs produced as intracellular nanorods by *Gayadomonas* sp. TNPM15 isolated from mangrove sediments and evaluate their impact on *F. oxysporum*, and *A. alternata*.

## 2. Materials and Methods

### 2.1. Sample Collection

Mangrove sediments were collected from Safaga Island, the Red Sea, Egypt (26°45′55.6″ N 33°57′38.3″ E) using sterile disposable wooden spatulas during low tides. The collected samples were placed in sterile Whirl-Pak^®^ sample bags (Nasco, Kellogg, ID, USA) and transported to the laboratory on ice.

### 2.2. Isolation of TeNPs-Producing Bacteria

Mangrove sediment samples were suspended in sterile seawater and vigorously vortexed for 15 min, and then serially diluted in sterile seawater. Thereafter, 100 μL of each dilution were plated onto tryptone yeast extract seawater (TYS) agar (tryptone 10 g, yeast extract 5 and agar 15 g/L natural seawater) plates supplemented with 1 mM potassium tellurite. After 24–72 h of incubation at 30 °C, black-colored colonies were picked as presumptive TeNPs-producing bacteria. The selected colonies were re-streaked on the same medium until discrete colonies were obtained. To ensure that the color was not an organic bacterial pigment, the selected colonies were re-streaked on TYS agar plates without potassium tellurite and monitored for black pigment production during the incubation period (24–72 h) at 30 °C. Furthermore, the formation of intracellular tellurium nanostructures was confirmed by transmission electron microscopy (JEM-1400, JEOL, Tokyo, Japan) operating at 80 KV, after negative staining of bacterial cells with 1% phosphotungstic acid (*w*/*v*).

### 2.3. Bacterial Identification

A promising TeNPs-producing strain designated TNPM15 was identified by amplification and sequencing of its 16S rRNA gene. Typically, the genomic DNA was extracted using a PureLink DNA Purification Kit (Thermo Scientific, Waltham, MA, USA) according to the manufacturer’s protocol. Genomic DNA was used as a template for the amplification of the 16S rRNA gene by polymerase chain reaction (PCR), using the universal primers 27F (5′-AGAGTTTGATCMTGGCTCAG-3′) and 1492R (5′-GGYTACCTTGTTACGACTT-3′). The PCR reaction was carried out by mixing 5 μL 10X Dream Taq buffer, 50 ng genomic DNA template, 0.4 μM of each primer, 0.2 mM of each dNTP, and one Unit Dream Taq DNA polymerase (Thermo Scientific, MA, USA), and, finally, nuclease-free water was added to increase the volume up to 50 μL. The amplification program contained an initial denaturation step at 95 °C for 4 min, followed by 30 cycles of denaturation at 95 °C for 40 s, annealing at 55 °C for 40 s, extension at 72 °C for 1.5 min and a final extension at 72 °C for 8 min in a ProFlex thermocycler (Applied Biosystems, Foster City, CA, USA). The amplified PCR products were purified using a GeneJET PCR purification kit (Thermo Scientific, MA, USA) according to the manufacturer’s protocol.

The nucleotide sequence of the purified PCR product was sequenced in both directions using the 27F and 1492R primers using an ABI 3730xI sequence analyzer (Applied Biosystems, USA). Subsequently, CodonCode aligner software v. 7.1.2 was used to assemble the forward and reverse DNA sequence reads. The sequence similarity search was performed at the National Center for Biotechnology Information (NCBI) server using BLASTN (http://www.ncbi.nlm.nih.gov/blast; accessed on 20 December 2022) and the EzTaxon-e server database [59]. The phylogenetic tree in the context of 16S rRNA gene sequence was constructed using the Neighbor-Joining method of the MEGA-X software (version 10.0.5) with a bootstrap analysis based on 1000 replicates.

### 2.4. Biosynthesis of Tellurium Nanoparticles

The biosynthesis of TeNPs was achieved by culturing the strain TNPM15 in TYS broth amended with 1 mM potassium tellurite at 30 °C for 24 h, with continuous shaking (180 rpm). Afterwards, the bacterial cells were harvested by centrifugation at 10,000× *g* for 20 min, followed by washing the pellet twice with sterile saline solution. The pellets were then resuspended in a lysis buffer containing 10 mM Tris-HCl (pH 8.3), 1 mM EDTA, 100 mM NaCl, 1% SDS (*w*/*v*), and 0.5% Triton X100 (*v*/*v*); and the bacterial cells were disrupted by sonication for 5 min at 100 W. Thereafter, the released TeNPs were purified by sequential centrifugation at 12,000× *g* for 15 min and washing with 1.5 M Tris-HCl buffer (pH 8.3) containing 0.5% SDS (*w*/*v*). Then, the purified TeNPs were washed three times with deionized water and, finally, the pellet was resuspended in ultrapure water (18.2 Mcm @25 °C) and sterilized by filtration through 0.45 µm syringe filters.

### 2.5. Characterization of Tellurium Nanoparticles

The size and shape of biogenic TeNPs were determined using transmission electron microscopy. In brief, a drop of the diluted suspension of the biogenic TeNP was loaded on a carbon-coated copper grid and air-dried, and observed using a TEM (JEM-1400, JEOL, Japan) operating at 80 KV. Scanning electron microscopy (SEM) analysis was performed using a Quattro S instrument (Thermo Scientific, MA, USA) equipped with energy-dispersive X-ray spectroscopy (EDX) to determine the chemical composition of the as-prepared nanostructures. The X-ray diffraction (XRD) was investigated by a D8 Discover diffractometer (Bruker, Karlsruhe, Germany) using Cu Kα radiation as a light source with a current of 30 mA and an applied voltage of 40 kV. The 2θ angles ranged from 20 to 60° with a scan speed of 0.5°/min. Raman spectra of the biogenic TeNPs were recorded using a LabRAM HR Evolution Raman spectrometer (HORIBA Ltd., Kyoto, Japan) equipped with an air-cooled open electrode 1024 × 256-pixel CCD detector. The TeNPs were placed as thin films on a glass slide and dried in air at ambient temperature. Normal Raman spectra were acquired using 532 nm excitation (spectral range 50–300 cm^−1^) with an acquisition interval of 20 s. Fourier-transform infrared (FTIR) spectroscopy was used to determine the functional groups. The scanning spectra were obtained in the range of 4000 to 400 cm^−1^ using a Nicolet 6700 FT-IR spectrometer (Thermo Scientific, MA, USA).

### 2.6. Fungal Phytopathogens

The antifungal effect of the biogenic TeNPs was investigated against two phytopathogenic fungi, *Fusarium oxysporum* AUMC 10313 and *Alternaria alternata* AUMC 3882, provided from the Assiut University Mycology Center (AUMC), Assiut, Egypt.

### 2.7. Germination Test

The inhibitory effect of the biogenic TeNPs on spore germination was evaluated in vitro. Briefly, *F. oxysporum* and *A. alternata* were cultured on potato dextrose agar (PDA) plates (Condalab, Madrid, Spain) at 28 °C for 7 days. Afterwards, spores were harvested, suspended in sterile saline solution (NaCl, 0.9%) and filtered through sterile muslin to remove mycelia. The spore count was adjusted to 2 × 10^6^ spores/mL using a hemocytometer. Then, 50 µL of spore suspension was added to each well of a 96-well microtiter plate containing 100 μL potato dextrose broth (PDB) (Condalab, Madrid, Spain) with different TeNPs concentrations. The fungicide mancozeb (Santa Cruz Biotechnology, Dallas, TX, USA) was added at various concentrations (10, 20, 30, 40 and 50 µg/mL) instead of the TeNPs as a positive control, and sterile saline solution was used as a negative control. The plates were incubated at 28 °C for 16 h for germination. Subsequently, 100 spores were analyzed with a DM500 optical microscope (Leica, Heerbrugg, Switzerland) and the percentage of germinated spores was calculated. A spore was considered germinated if the germ tube length is equal to or longer than the spore length. The minimum inhibitory concentration (MIC) was determined as the lowest concentration of biogenic TeNPs that completely inhibits spore germination. The MIC values were expressed as µg/mL and calculated from the regression analysis curves.

### 2.8. Spore Morphology and Ultrastructure Analysis

The probable morphological changes and ultrastructural damage of fungal spores exposed to TeNPs were observed by using TEM. In brief, spore suspensions of *F. oxysporum* and *A. alternata* were prepared as above and exposed to TeNPs at the half concentration of their corresponding MIC values. Control groups were prepared using the same procedure, but TeNPs were not incorporated. After incubation at 28 °C for 3 h, the spores were collected by centrifugation at 6000× *g* for 10 min, washed twice with sterile saline and fixed with 3% (*v*/*v*) glutaraldehyde for 4 h overnight, then post fixed with 1% (*w*/*v*) osmium tetroxide at 4 °C for 3 h. Then, samples were sequentially dehydrated using a graded ethanol series (20 to 100%). Subsequently, the dehydrated spores were placed into an embedding plate, then permeated with pure propylene oxide, then 100% Spurr. Finally, ultrathin sections were prepared with a microtome and observed at 80 kV using a JEM-1400 transmission electron microscope (JEOL, Japan). Ultrastructural alterations of the spores were compared with untreated controls to assess the effect of TeNPs against spores of *F. oxysporum* and *A. alternata*.

### 2.9. Leakage of Proteins and DNA

To determine the influence of TeNPs on membrane integrity, the leakage of proteins and DNA from exposed phytopathogens was assessed. In brief, spore suspension of *F. oxysporum* and *A. alternata* (10^6^ spore/mL) were incubated for 24 h at 28 °C with TeNPs at the half MIC values. Untreated spore suspension sets (without TeNPs) served as controls. At the end of incubation period, cultures were centrifuged at 10,000 rpm for 15 min at 4 °C and the supernatants were collected to measure the extracellular proteins and DNA. Subsequently, leakage of proteins and DNA upon exposure to the biogenic TeNPs was investigated following the method described by Khalil et al. [60].

### 2.10. Impact of TeNPs on Plant Cell Wall-Degrading Enzymes (CWDEs)

The potential inhibitory effect of TeNPs on the CWDEs of *F. oxysporum* and *A. alternata* was investigated. We focused on the influence of TeNPs on essential hydrolases associated with their pathogenicity as cellulase, pectinase and xylanase. To determine the activities of the CWDEs, each phytopathogen was inoculated into an induction broth [(g/L): NaNO_3_ 2; K_2_HPO_4_ 1; KCl 0.5; MgSO_4_ 7H_2_O 0.5; FeSO_4_ 7H_2_O 0.01] amended with 1% carboxymethyl cellulose (CMC), pectin or xylan for cellulase, pectinase and xylanase, respectively. All treated media were supplemented with TeNPs at the half concentration of their corresponding MIC values. Control groups were prepared using the same procedure, but TeNPs were not incorporated. After incubation at 28 °C for 7 days, cellulases, pectinases and xylanases were determined by measuring the amounts of reducing sugar released from 1% CMC, pectic acid and xylan by 3,5-dinitrosalicylic acid (DNSA) method using glucose, D-galacturonic acid and xylose as a standard, respectively [61]. Finally, the enzymatic activities were compared with those of untreated controls.

### 2.11. Cytotoxicity Assay

The Human Skin Fibroblast (HSF) cell line, obtained from Nawah Scientific Inc. (Mokatam, Cairo, Egypt), was used for the cytotoxicity investigations. The Dulbecco’s Modified Eagle medium (DMEM) supplemented with 10% heat-inactivated fetal bovine serum (Gibco; Thermo Fisher Scientific, Loughborough, UK) and antibiotics (100 µg/mL streptomycin and 100 units/mL penicillin) was used for culturing and maintenance of the cell line. The HSF cells were cultured in a water-jacketed incubator (BINDER GmbH, Tuttlingen, Germany) at 37 °C under 5% CO_2_ in a high humidity atmosphere.

The cytotoxicity of TeNPs against HSF cells was assessed using the sulforhodamine B (SRB) assay. Briefly, aliquots of 100 μL cell suspension (5 × 10^3^ cells) were added to a 96-well microtitre plate, and various concentrations of TeNPs were added and incubated for 72 h. In another set, untreated HSF cells served as control. Afterwards, the media was replaced with 150 μL of 10% trichloroacetic acid (TCA) solution and incubated at 4 °C for 1 h. Subsequently, the TCA solution was discarded and the plates were gently washed five times with distilled water and allowed to dry. Aliquots of 70 μL SRB solution (0.4% *w*/*v* in 1% glacial acetic acid) were added and incubated in the dark at room temperature for 10 min. Thereafter, plates were washed thrice with 1% acetic acid and allowed to air-dry again. Then, the protein-bound SRB stain was dissolved in 150 μL of Tris buffer (10 mM, pH 10.5) and the absorbance was measured at 540 nm using Epoch2 microplate spectrophotometer (BioTek, Santa Clara, CA, USA).

### 2.12. Statistical Analysis

The measured data were subjected to the analysis of variance (ANOVA) appropriate to the design. The significant differences between treatments were compared with the critical difference at 5% level of probability by the Duncan’s test using IBM SPSS software version 22.

## 3. Results

### 3.1. Isolation of TeNPs-Producing Bacteria

**Figure 1:**
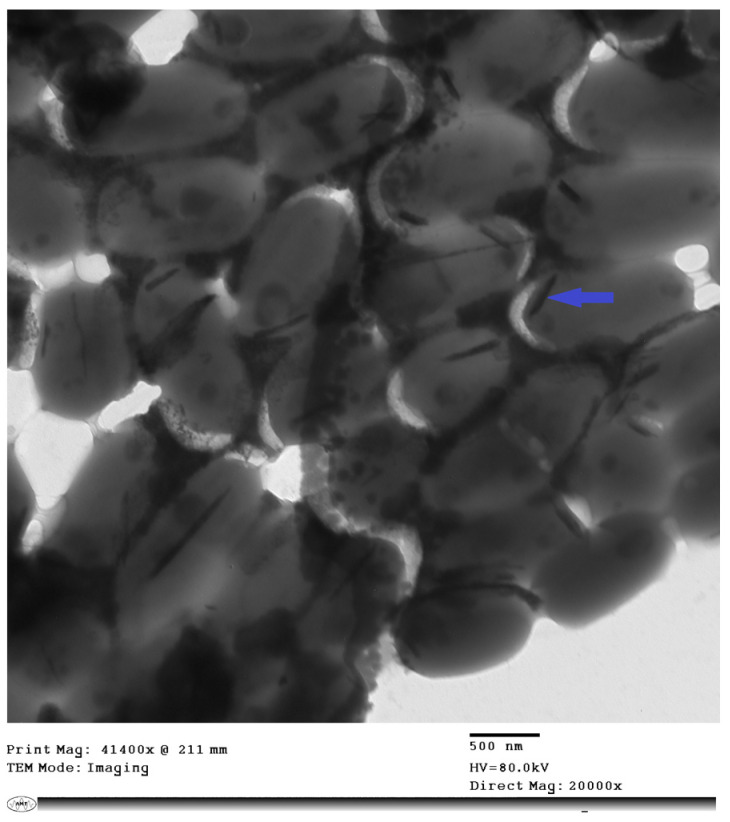
TEM micrograph showing intracellular formation of nanorods by *Gayadomonas* sp. TNPM15. The blue arrow indicates the formation of intracellular rod-shaped nanostructure.

**Figure 2:**
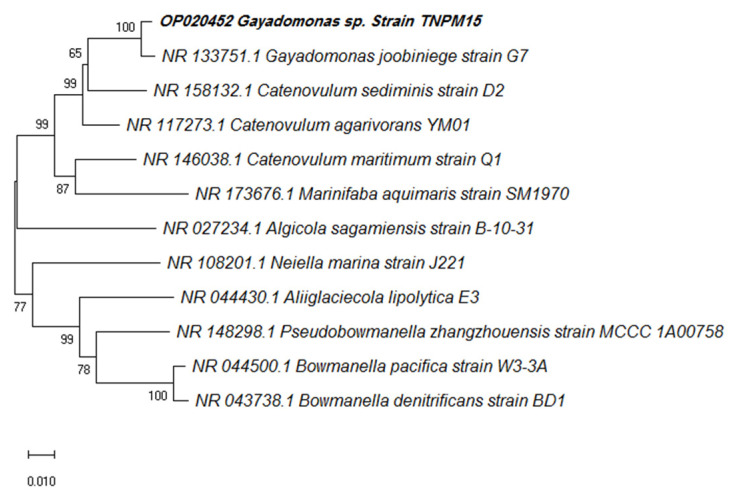
Phylogenetic tree showing the relationships between *Gayadomonas* sp. TNPM15 and the most closely related species.

Several strains isolated from the mangrove sediments were able to grow in the presence of 1 mM potassium tellurite. The reduction of Te (IV) to Te^(0)^ by bacterial isolates was detected by the black-colored precipitate in the culture, indicating the reduction of tellurite to black Te^(0)^ precipitate. Black precipitates were not detected in cultures grown on the same medium without emendation, confirming the production of elemental tellurium rather than organic pigmentation. Furthermore, TEM analysis confirmed the production of intracellular rod-shaped nanostructures by a bacterial strain designated TNPM15 exhibiting a characteristic black color in the presence of tellurite (Figure 1). The selected strain TNPM15 was identified as *Gayadomonas* sp. based on its 16S rRNA gene sequence (accession number OP020452). BLAST results revealed that the strain TNPM15 shares 98.92% identity with *Gayadomonas joobiniege* strain G7 (accession number NR_133751.1) and 96.32% with *Catenovulum agarivorans* YM01 (accession number NR_117273.1). The Neighbor-Joining phylogenetic tree in the context of 16S rRNA gene sequence was constructed (Figure 2).

### 3.2. Biosynthesis and Characterization of TeNPs

**Figure 3:**
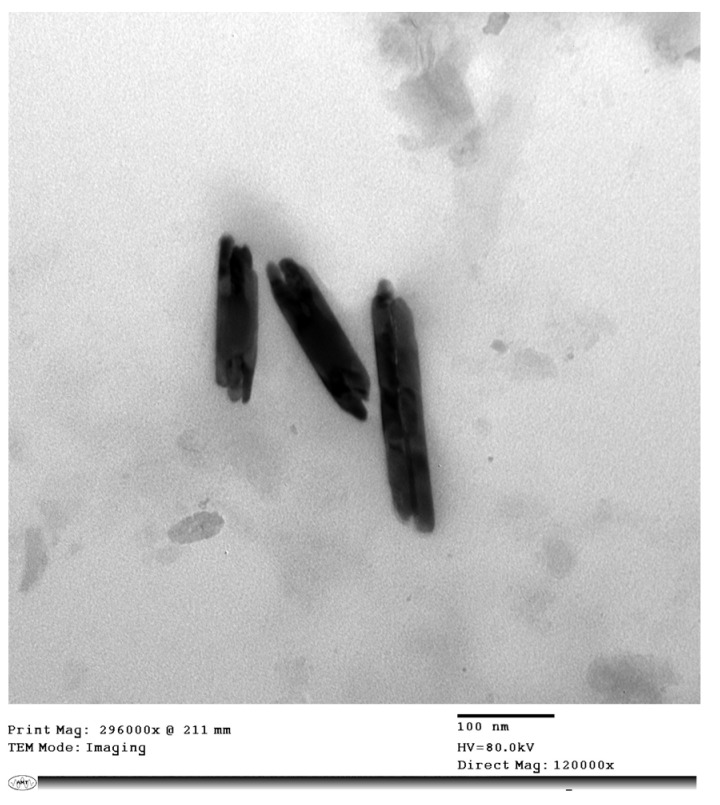
Transmission electron micrograph showing the rod-shaped biogenic TeNPs produced by *Gayadomonas* sp. TNPM15.

**Figure 4:**
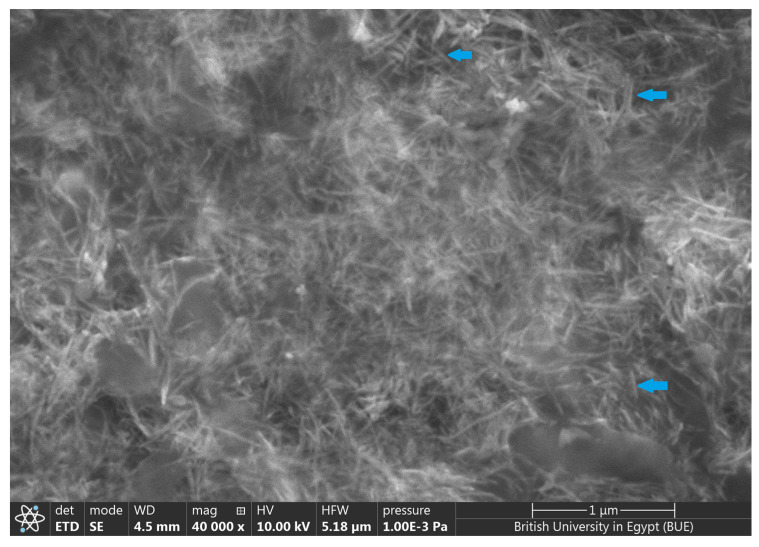
Scanning electron micrograph showing the rod-shaped biogenic TeNPs produced by *Gayadomonas* sp. TNPM15. The blue arrows indicate the formation of rod-shaped nanostructures.

**Figure 5:**
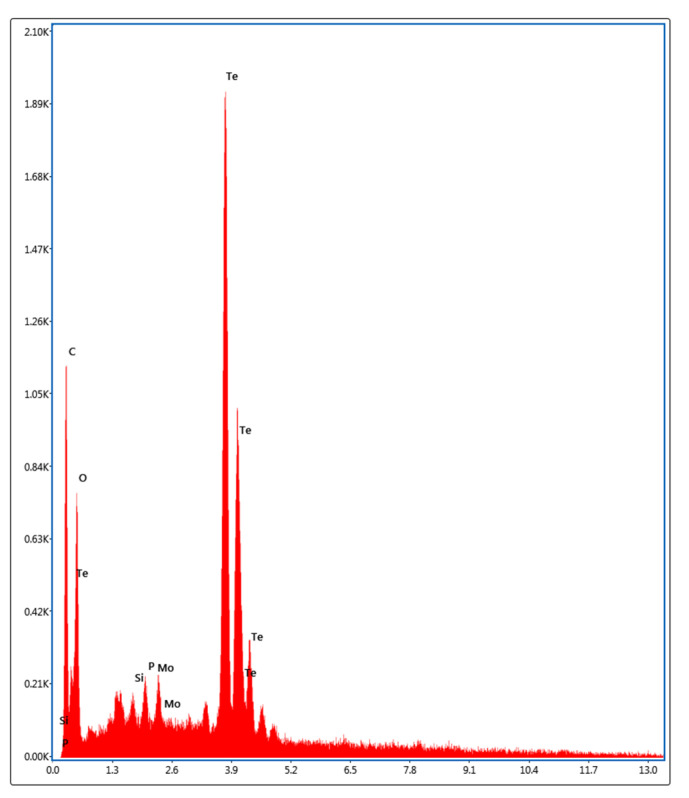
EDX spectrum of the biogenic TeNPs produced by *Gayadomonas* sp. TNPM15.

**Figure 6:**
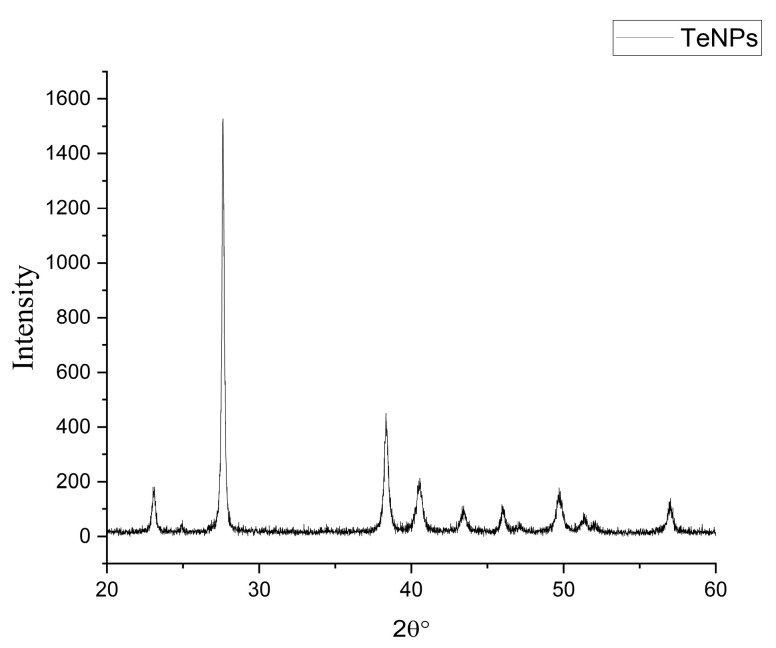
XRD spectrum of the biogenic TeNPs produced by *Gayadomonas* sp. TNPM15.

**Figure 7:**
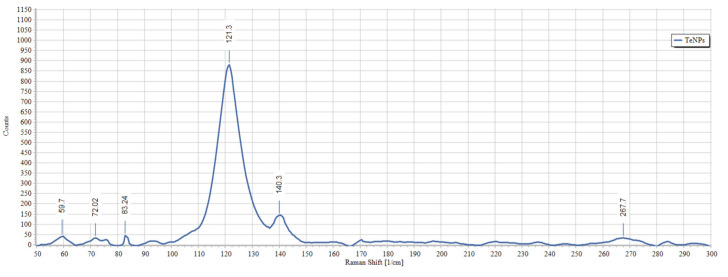
RAMAN spectrum of the biogenic TeNPs produced by *Gayadomonas* sp. TNPM15.

**Figure 8:**
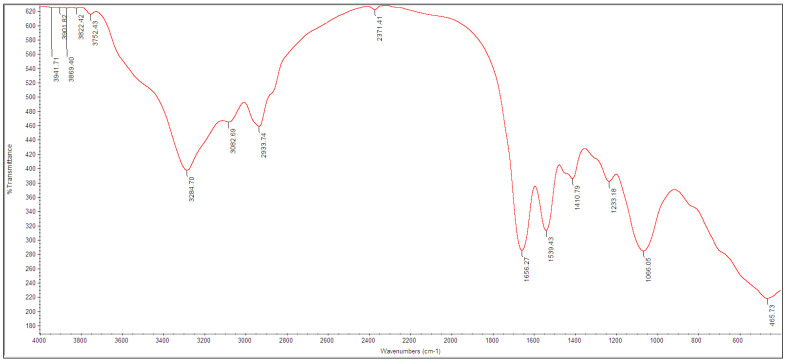
FTIR spectrum of the biogenic TeNPs produced by *Gayadomonas* sp. TNPM15.

Biogenic TeNPs were produced by exploiting the tellurite-reduction capability of *Gayadomonas* sp. strain TNPM15 cultured in TYS broth supplemented with potassium tellurite. The produced intracellular TeNPs were recovered and resuspended in ultrapure water after purification steps. Electron microscopy was employed to clarify the size and shape of the purified nanoparticles. The TEM demonstrated that the purified TeNPs appeared as nanorods with a diameter range from 15 to 23 nm and different lengths reaching up to 243 nm (Figure 3). Moreover, SEM observation clearly confirmed the formation of rod-shaped nanostructures (Figure 4). Based on the results of elemental analysis by EDX, we evidenced that the black biogenic nanostructures were mainly composed of tellurium (Figure 5). Furthermore, the XRD spectrum revealed the crystalline nature of the produced TeNPs (Figure 6). Additionally, Raman spectra appeared in the whole lower-frequency region, showing characteristic vibration peaks attributed to tellurium at 121.3 and 140.3 cm^−1^, affirming the formation of TeNPs (Figure 7). In addition, the FTIR spectrum provided insights into the functional groups incorporated into the surface of the as-prepared biogenic tellurium nanostructures (Figure 8).

### 3.3. Germination Test

**Figure 9:**
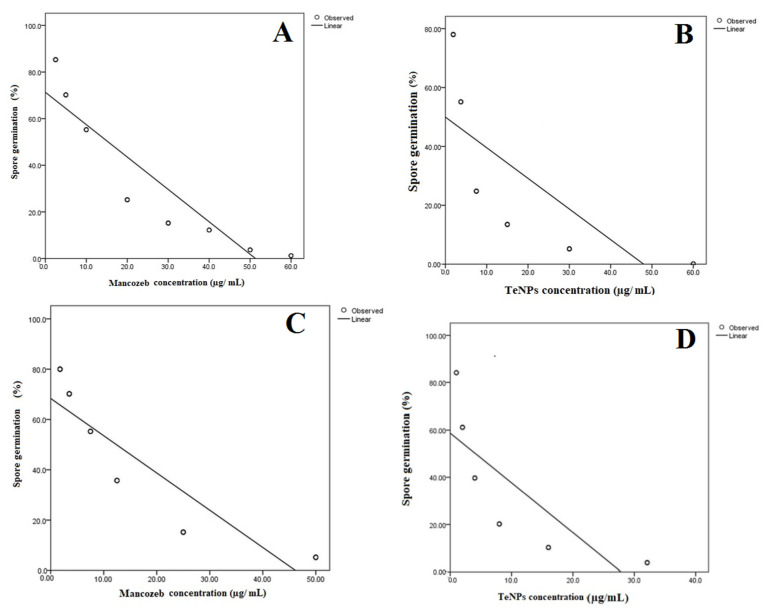
Linear regression model fit of spore germination. (**A**) *F. oxysporum* treated with mancozeb, (**B**) *F. oxysporum* treated with TeNPs, (**C**) *A. alternata* treated with mancozeb, (**D**) *A. alternata* treated with mancozeb.

The antifungal activity of the biosynthesized TeNPs was assessed against the phytopathogenic fungi *F. oxysporum* and *A. alternata.* Our results proved the ability of the biogenic TeNPs to inhibit fungal spore germination. The MIC values which completely inhibited spore germination of *F. oxysporum* and *A. alternata* were statistically deduced from the linear regression model fit (Figure 9). In the case of *F. oxysporum*, the deduced MIC were 48.1 and 38.0 µg/mL for TeNPs and mancozeb, respectively. On the other hand, TeNPs and mancozeb had MIC values of 27.6 and 30.5 µg/mL against *A. alternata,* respectively.

### 3.4. Spore Morphology and Ultrastructure Analysis

**Figure 10:**
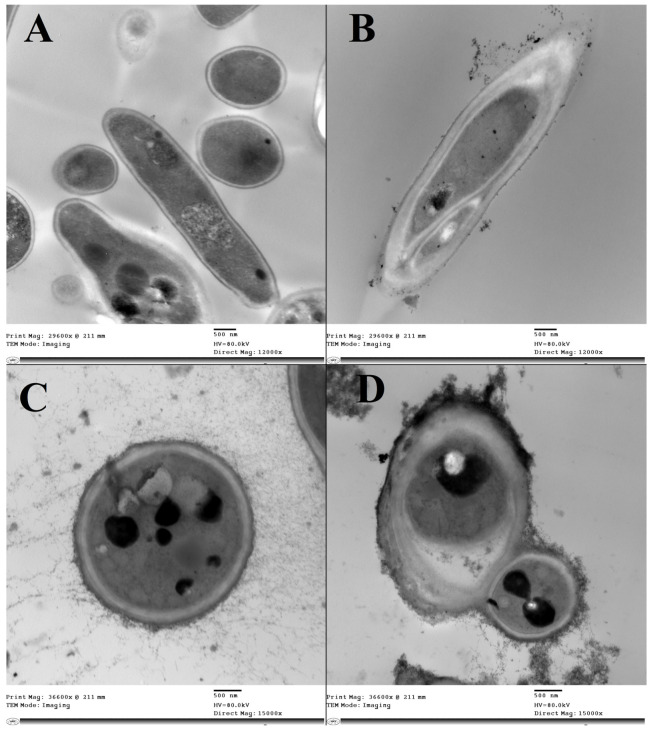
TEM micrographs showing the ultrastructure alternations in fungal spores upon exposure to the biogenic TeNP produced by *Gayadomonas* sp. TNPM15. (**A**) Untreated *F. oxysporum* (Control), (**B**) TeNPs-treated *F. oxysporum*, (**C**) Untreated *A. alternata* (Control), (**D**) TeNPs-treated *A. alternata*.

The ultrastructural analysis of *F. oxysporum* and *A. alternata* spores was investigated by electron microscopy. Generally, spores in control experiments appeared intact with well-defined ultrastructure and smooth cell walls, whereas those exposed to biogenic TeNP exhibited prominent ultrastructure alternations (Figure 10). TEM observations indicated that the treated fungal spores seem to be deformed with ruptured walls in both investigated phytopathogenic fungi. Results indicated more deformation in the case of *A. alternata* than in *F. oxysporum*.

### 3.5. Leakage of Proteins and DNA

**Figure 11:**
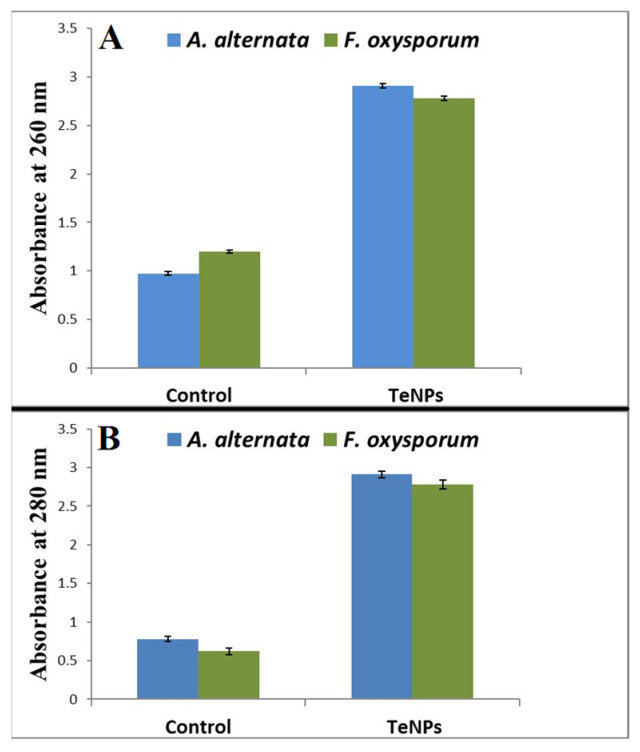
Effect of the biogenic TeNPs produced by *Gayadomonas* sp. TNPM15. on the leakage of proteins (**A**) and DNA (**B**) from spore suspension of *F. oxysporum* and *A. alternata*.

To gain further insight into the antifungal activity of TeNPs, we studied the probable leakage of proteins and DNA in TeNPs-treated spores compared with untreated control. The results revealed a significant increase in extracellular protein and DNA levels, indicating increased permeability of spore membrane and leakage of an intracellular substance (Figure 11). Likewise, TeNPs were more effective against *A. alternata* than *F. oxysporum* in the disruption of spore membranes.

### 3.6. Impact of TeNPs on Plant Cell Wall-Degrading Enzymes

Application of the TeNPs to the growth medium at half of their MIC values resulted in significant inhibition of cellulase, pectinase, and xylanase activities in both investigated phytopathogenic fungi (Table 1). The results demonstrated about 78.2 and 84.9% inhibition of cellulase activity in treated *F. oxysporum* AUMC 10313 and *A. alternata* AUMC 3882, respectively, whereas the biogenic TeNPs reduced their pectinase activity by 51.3 and 59.3%, respectively. On the other hand, TeNPs exerted a relatively tenuous inhibitory effect against xylanase activity compared with that against cellulase and pectinase.

### 3.7. Cytotoxicity Assay

In the present study, the cytotoxicity and biocompatibility of the bacterial-mediated biogenic TeNPs were tested, in vitro, against HSF cell lines. Indeed, the SRB assay indicated that the biogenic TeNPs showed no significant cytotoxic effect towards HSF cells at concentrations up to 80 μg/mL. The results revealed that the half-maximal inhibitory concentration (IC_50_) value of the biofabricated TeNPs against HSF cells was 125 μg/mL.

## 4. Discussion

The exploitation of the biomineralization potential of some tellurite-resistant bacteria in the bioconversion of chalcogen oxyanions is considered a valuable route to develop green strategies to fabricate unique nanoscale materials. In this investigation, the tellurite-resistant marine bacterium, *Gayadomonas* sp. TNPM15, was isolated from mangrove sediments on a tellurite-containing culture media. The appearance of the black color was the initial indication of the mineralization of tellurite to elemental tellurium, and TEM observations confirmed the formation of intracellular TeNPs. Mangrove has been reported as a highly productive ecosystem with noteworthy microbial diversity [62,63]. Likewise, the tellurite-resistant *Raoultella* sp. strain WAY isolated from wastewater reduced the Te(IV) forming elemental tellurium as black colonies exhibited the potentiality for the green synthesis of tellurium nanorods [64]. Previous studies have reported the isolation of tellurite-reducing bacteria that produce TeNPs from various natural habitats [65,66,67,68].

In our study, the biogenic TeNPs were produced as intracellular nanorods. These intracellular nanostructures were recovered following the lysis of bacterial cells by sonication. The size and shape of the purified bioinspired nanostructures were determined by TEM and SEM, which confirmed the production of nanorods. Furthermore, EDX analysis revealed that the prepared nanorods were composed mainly of tellurium, in which organic compounds were incorporated due to the biosynthesis process. The presence of organic matter, including proteins in the biogenic TeNPs, was evidenced by FTIR analysis. Bands at 1656 cm^−1^ and 1539 cm^−1^ are mainly associated with a C-O stretching peptide bonds from amide I and N-H bending and C-N stretching vibration from amide II, respectively [69,70,71]. The band at 2933 cm^−1^ donates the C-H asymmetric stretching of alkanes [72]. In addition, the peak at 1053 cm^−1^ could correspond to the C–N stretching vibration of the amine, while the band at 1410 cm^−1^ corresponds to the N–H bending of primary amides due to carbonyl stretch in proteins [73]. It has been suggested that proteins may be involved in the biosynthesis of various metal nanoparticles as reducing and capping agents that stabilize the biogenic nanoparticles. In agreement with our study, the FTIR spectrum of silver nanoparticles by *Penicillium* sp. revealed bands at 1644, 1538, and 3290 cm^−1^ correspond to the binding vibrations of amide I and amide II band of protein, respectively [74]. These results are in harmony with previous investigations reporting the presence of organics such as proteins on the surface of various biogenic nanoparticles as capping and stabilizing agents [19,54,75,76]. Furthermore, the nanoparticles were characterized by XRD, and their pattern confirmed that *Gayadomonas* sp. TNPM15 produced crystalline nanoparticles of elemental tellurium nanoscale hexagonal crystal structure [77,78]. In addition, Raman spectra revealed the appearance of the specific peaks attributed to the A1 and E phonon modes of vibration for tellurium [79,80]. The biosynthesis of tellurium nanorods by *Gayadomonas* sp. TNPM15 supports the findings of previous works that demonstrated the biosynthesis of rod-shaped TeNPs by various microorganisms [2,81,82,83]. Our findings agree with most previous literature reporting the intracellular biosynthesis of TeNPs [25,26,75,84]. On the other hand, the extracellular biosynthesis of TeNPs is infrequently reported using certain bacterial species such as *Rhodobacter capsulatus* and *Shewanella oneidensis* [22,85].

The findings of the current study illustrate the potent antifungal effect of the biogenic TeNPs-produced *Gayadomonas* sp. TNPM15 against two important fungal phytopathogens. Practically, the biogenic tellurium nanorods inhibited the spore germination of both investigated fungi and were evidenced to cause significant malformation and ultrastructural alternation on fungal spores by electron microscopy. Due to the rupture of cell walls and disintegration of membranes, the spores lost their intracellular content. This disintegration of the membrane was affirmed by increasing in the leakage of DNA and proteins. These findings concord with previous reports, suggesting that the antimicrobial effect of various nanoparticles may be attributed to the disintegration of membranes and leakage of the intracellular content, as well as the rupture of cell walls [2,86,87,88,89,90,91]. Interestingly, the biogenic TeNPs produced by *Gayadomonas* sp. TNPM15 were more effective than mancozeb against *A. alternata*, and their antifungal activity is more or less comparable to the mancozeb (positive control) against *F. oxysporum*. Mancozeb is a broad-spectrum fungicide that is used worldwide for fungal disease control. For decades, it has been an invaluable tool to farmers all over the world faced with the challenge of controlling fungal pests in their crops [92]. Likewise, several previous studies used mancozeb as a standard positive control for evaluating the antifungal efficacy of various biological and chemical antifungal agents [69,93,94]. In addition to their direct antifungal effects, TeNPs caused a remarkable inhibition of the plant cell wall-degrading enzymes. This inhibitory potential against cellulase, pectinase, and xylanase could result in the attenuation of the virulence of the exposed phytopathogens. Cellulose, hemicellulose, lignin, and pectin are the most common polysaccharides in plant cell walls. Thus phytopathogens secrete several extracellular enzymes responsible for cell wall degradation such as cellulase, pectinase, and xylanase to facilitate their invasion, resulting in adverse effects on the infected plants [95]. Accordingly, attempts to inhibit the cell wall, degrading enzymes secreted by phytopathogens, may restrict and attenuate their invasion, colonization, and expansion. Regarding the potential cytotoxicity, the cytotoxic effects of the biogenic TeNPs produced by *Gayadomonas* sp. TNPM15 were assessed in vitro against HSF cells. It is worth mentioning that TeNPs exhibited excellent biocompatibility towards HSF cells with no apparent toxicity at concentrations up to 80 μg/mL. By comparing the effective antifungal concentrations of TeNPs produced by *Gayadomonas* sp. TNPM15 with the IC_50_ value (125 μg/mL) against HSF cells, it can be concluded that the biogenic TeNPs produced by *Gayadomonas* sp. TNPM15 could be used as a safe non-toxic antifungal agent against phytopathogenic fungi.

## 5. Conclusions

The antifungal potential of the biogenic rod-shaped TeNPs produced by *Gayadomonas* sp. TNPM15 provides a promising solution to combat fungal infections such as root rot and black spot diseases caused by *Fusarium* and *Alternaria* spp., respectively. The biosynthesized TeNPs induce prominent ultrastructure alternations such as deformation and damage in the cell wall. Furthermore, the antifungal potential of the biogenic TeNPs is attributed to their inhibitory effect of fungal spore germination and disruption of membrane permeability and integrity, as evidenced by TEM and increased leakage of the intracellular components, in addition to their inhibitory effects on plant cell wall-degrading enzymes. The cytotoxicity assessments revealed their good biocompatibility and low toxicity toward HSF cells. We concluded that the biogenic tellurium nanorods produced by *Gayadomonas* sp. TNPM15 are a potential candidate as a robust antifungal with non-toxic doses.

## Figures and Tables

**Table 1:** Impact of the biogenic TeNPs produced by *Gayadomonas* sp. TNPM15 on the plant cell wall-degrading enzymes of *F. oxysporum* AUMC 10313 and *A. alternata* AUMC 3882.

Phytopathogen	Relative Enzyme Activity (%)		
	Cellulase	Pectinase	Xylanase
*F. oxysporum* AUMC 10313	21.8 ± 2.4	48.7 ± 1.6	72.1 ± 1.1
*A. alternata* AUMC 3882	15.1 ± 2.7	40.7 ± 1.9	70.7 ± 1.3

## Data Availability

The essential data supporting the reported results are contained in this study. All other data are available on request from the corresponding authors.
